# Spatial analysis of hypospadias cases in northern France: taking clinical data into account

**DOI:** 10.1186/s12887-020-02332-1

**Published:** 2020-09-21

**Authors:** Arthur Lauriot Dit Prevost, Michael Genin, Florent Occelli, René-Hilaire Priso, Remi Besson, Caroline Lanier, Dyuti Sharma

**Affiliations:** 1 CHU Lille, Clinique de Chirurgie et Orthopédie de l’Enfant, F-59000 Lille, France; 2 CHU Lille, Centre de référence du développement génital DEV-GEN, F-59000 Lille, France; 3 Univ. Lille, CHU Lille, ULR 2694 METRICS – Évaluation des technologies de santé et des pratiques médicales, F-59000 Lille, France; 4 Univ. Lille, Laboratoire de Génie Civil et géo-Environnement, F-59000 Lille, France; 5 Faculté ILIS/Faculté de pharmacie de Lille – LSVF, Lille, F-59000 France

**Keywords:** Birth defect, Congenital malformation, Spatial cluster detection, Geographical analysis, Ecological regression, Endocrine-disrupting chemicals, Deprivation index

## Abstract

**Background:**

Strong evidence for a causal role of environmental factors in a congenital anomaly is still difficult to produce. The collection of statistical data is crucial for gaining a better understanding of the epidemiology and pathophysiology of these anomalies. We aimed to evaluate spatial variations in hypospadias within our region and it’s association to socioeconomic and ecological factors, taking clinical data into account.

**Methods:**

All boys with hypospadias born in northern France and seen in Lille University Medical Center (Lille, France) between 1999 and 2012 were included in the analysis. We retrospectively collected geographic data, clinical data (especially known confounding factors associated with an elevated risk of hypospadias), and demographic, socio-economic and ecological data. We analyzed the entire study population and subsequently the subset of boys lacking confounding factors.

**Results:**

The study sample of 975 cases of hypospadias over the 13-year period resulted in an incidence of 25.4/10,000 male births, and was characterized by significant spatial heterogeneity (*p* < 0.005) and autocorrelation (*p* < 0.001). We detected two high-incidence clusters that differed with regard to their land use. After the exclusion of 221 patients with confounding factors, two high-incidence clusters with significant disease risks (1.65 and 1.75, respectively; *p* < 0.001) and a significant difference in land use (*p* < 0.001) again appeared. The first cluster contained a higher median [interquartile range] proportion of artificialized land (0.40 [0.22;0.47]) than the remaining “neutral areas” (0.19 [0.08;0.53]) did (*p* < 0.001). Conversely, the second cluster contained a higher median proportion of rural land (0.90 [0.78;0.96]) than the “neutral areas” (0.81 [0.47;0.92]) did (*p* < 0.001). The median deprivation index was significantly lower in the urban cluster (0.47 [0.42;0.55]) and significantly higher in the rural cluster (0.69 [0.56;0.73]) (*p* < 0.001).

**Conclusions:**

Our results evidenced the heterogeneous spatial distribution of cases of hypospadias in northern France. We identified two clusters with different environmental and social patterns – even after the exclusion of known confounding factors.

## Background

Hypospadias is one of the most frequent malformations of the genital organs in boys with a prevalence of 18/10,000 births in Europe. A significant variation from one country to another is observed, and also over time [1, 2]. It appeared to rise in the 1970s and 1980s but has remained stable since then [3].

The suspected causal factors include genetic factors, [4–7] iatrogenic factors (e.g. medications taken during pregnancy [8–10] or conception via assisted reproductive technologies (ART) [11, 12]) and environmental factors (e.g. fetal exposure to endocrine-disrupting chemicals or other chemical hazards) [13–17]. Hence, the collection of statistical data on all the potential genetic and environmental causal factors is crucial for gaining a better understanding of the epidemiology and pathophysiology of hypospadias and, ultimately, for guiding public health measures capable of mitigating exogenous risk factors to the greatest extent possible [5, 18]. Given that many environmental factors are ubiquitous and not easily measurable, strong evidence for a causal role in a congenital anomaly is still difficult to produce. Spatial analysis has emerged as a relevant, efficient tool for epidemiological research, [19–22] and has already been used to describe and evaluate spatial disparities in the occurrence of birth defects. Few studies have analyzed the spatial distribution of cases of hypospadias. The statistical methodology and the result of these studies varies [16, 23–28]. Most of the studies involving spatial analysis used massive administrative datasets from national registries; the latter did not contain much clinical information.

The aim of the present study was to (i) evaluate spatial variations in hypospadias incidence in northern France, (ii) evaluate potential associations between hypospadias and ecological variables, and (iii) compare spatial clusters of hypospadias from 1999 to 2012. The study was designed to take into account medical information regarding possible confounding variables We conceived this study as a new way of consolidating previous findings on the association between hypospadias and environmental factors.

## Methods

### Study area and data sources

The former *Nord - Pas-de-Calais* region of northern France comprises around 4,100,000 inhabitants over 12,414 km^2^ (326 inhabitants/km^2^). The region is distributed into 170 rural, industrial or urban *cantons* (a French local administrative unit). Lille University Medical Center (Lille, France) is the region’s tertiary referral center for pediatric surgery; surgical management of some congenital malformation were centralized in our hospital and all referred cases of hypospadias are seen and treated by a single pediatric urologist in our team. Furthermore, hypospadias consultations are organized in three different general hospitals across the region. Based on this surgical registry, we collected data about all consecutive cases of hypospadias between January 1999 and December 2012. All types of hypospadias were included - even those not requiring surgery. The study’s data collection procedures were registered with and validated by our University Medical Center’s Data Protection Officer (reference: DEC19–084).

All the data were retrospectively collected by manually reviewing each patient’s health records:

- The zip code at birth was collected in cases where the patient was born in Lille University Medical Center; otherwise the zip code noted during the first medical consultation was used. Patient were excluded if they had been adopted or had been born outside our region.
- The type of hypospadias was evaluated.
- Information was collected regarding potential confounding factors (CFs) associated with a higher risk of hypospadias: family history of hypospadias, [4, 29] syndromic association, consanguinity, known genetic defect, [6] ART, [11, 12, 30] vegetarian diet, [31] and known exposure to endocrine disrupting chemicals. Based on the French reference center for teratogenic agents, [32] and data from the literature we also considered the following medication -taken during pregnancy- as associated with a higher risk of hypospadias: anti-epileptic drugs such as valproate, gabapentin, clonazepam, primidone, topiramate, [8–10] diethylstilbestrol, [33] and thyroxine [34].
- Pregnancy-related data was also gathered such as intrauterine growth retardation, preterm delivery, and multiple pregnancy.

As a reference, the number of male births in each *canton* during the study period was extracted from data provided by the French National Institute of Statistics and Economic Studies (*Institut National de la Statistique et des Etudes Economiques, INSEE).*

Land use data were obtained from the European CORINE Land Cover database [35]. The proportion of artificial surfaces (level 1) and rural areas were calculated for each *canton*. In the rural land use category, we also considered agricultural surfaces in more detail (level 2).

The French Ecological Deprivation Index (EDI) was computed for each *canton* [36]. This deprivation index is composed of 10 variables: overcrowding, no access to central or electric heating, non-homeowner, unemployment, foreign nationality, no access to a car, unskilled work/farm work, a household with more than 6 people, a low educational level, and a single-parent household. The higher the *canton*’s EDI, the higher the level of deprivation. Socioeconomic data were extracted from the 2009 French national census (*INSEE*). Lastly, the EDI was considered in quartiles for ecological regression, as described in the “statistical analysis” section below.

Distance to the closest waste incineration plant (CWIP). Data from the French National Environmental Agency (*Agence de l’Environnement et de la Maîtrise de l’Energie*) were used to identify the region’s waste incineration plants operating before and during the study period. The Euclidian distance (in km) between each *canton*’s centroid and the CWIP was computed and considered in quartiles for the ecological regression.

### Statistical analysis

Quantitative variables were described as the mean (standard deviation) when normally distributed or as the median [interquartile range (IQR)] if not. The normality of distribution was assessed using histograms, a normal probability plot, and the Shapiro-Wilk test. Qualitative variables were described as the frequency (percentage).

We used the Potthoff-Whittinghill test to determine the presence of spatial heterogeneity in the incidence of hypospadias between spatial units [37]. The presence of spatial autocorrelation among spatial units was quantified using Moran’s index (with a value above 0 indicating the presence of autocorrelation) and probed using Moran’s test. The notion of spatial autocorrelation refers to the fact that geographically close spatial units tend to have similar values (incidence of hypospadias in our case) [38, 39].

The spatial distribution of hypospadias incidence was assessed by calculating the standardized incidence ratio (SIR). For each spatial unit, the SIR was defined as ratio between the observed number of cases and the expected number of cases. Given that the SIRs were unstable (due to low frequencies and spatial autocorrelation), the ratios were smoothed using the Bayesian Poisson regression model developed by Besag et al. [40]

Associations between the incidence of hypospadias and ecological variables (considered in quartiles) were assessed using an extension of the previous model, namely ecological regression (i.e. the inclusion of ecological covariates as fixed effects). For each covariate, the relative risk (RR) of hypospadias incidence and its 95% Bayesian credibility interval (BCI) were computed.

The detection of significant spatial clusters of a high hypospadias incidence was performed with isotonic spatial scan statistics based on a Poisson model [21, 41]. An isotonic regression function is used to model the potential cluster, since the high risk of hypospadias in a spatial cluster is not considered constant, but modelled by a piecewise decreasing risk function: the risk decreases as the distance from the cluster center to its boundaries increases, and the function takes a step down (isotonic levels) at several locations. The risk function is fitted with an isotonic regression, and no a priori assumptions about the number of steps are made. These techniques make it possible to detect clusters with an epicenter where the risk is the higher. The significance of each detected cluster was been evaluated in 9999 Monte-Carlo replications under the null hypothesis of the absence of clusters; In the context of scan statistics, Monte-Carlo methods consist of simulating a large number of data sets under the null hypothesis of absence of clusters to give an approximation of the distribution of the test statistic, thus making it possible to calculate a *p*-value for the spatial clusters detected on the real data. The RR was calculated for each significant cluster and each isotonic level, and was interpreted as the risk of observing hypospadias inside the cluster, relative to the risk outside the cluster.

In order to compare clusters based on ecological data, the *cantons* were categorized by cluster (i.e. the *cantons* composing each identified cluster). A “neutral” group of cantons comprised those which do not fall inside a cluster. For the comparison of clusters on the basis of clinical data at the individual patient level, the same groups were considered but with regard to the patient’s *canton* of residence.

Intergroup comparisons of quantitative variables were performed with a one-way ANOVA (if appropriate) or a non-parametric Kruskal-Wallis’ test. Chi-square tests were used for qualitative variables. The Potthoff-Whittinghill test, Moran’s test, SIR smoothing, ecological regressions, and comparisons were carried out using R software (version 3.4.3) and the latter’s DCluster and R-INLA packages [42]. Maps were produced using QGIS software (version 2.18) [43]. The threshold for statistical significance was set to *p* < 0.05.

We performed spatial analyses on two distinct layers. Layer #1 comprised the whole study population (i.e. all boys with hypospadias born within our region, excluding those born outside the region or who had been adopted). Layer #2 comprised the subset of boys with hypospadias and no known potential causal factors (or potential confounding factors) for hypospadias, which might otherwise have biased our analysis of environmental factors The exclusion criteria for the layer #2 analysis was the presence of at least one CF as described in 2.1.

The clusters comparison is only described for the second layer in this article (for the first layer of the spatial analysis, see the Additional file 2).

## Results

**Fig. 1:**
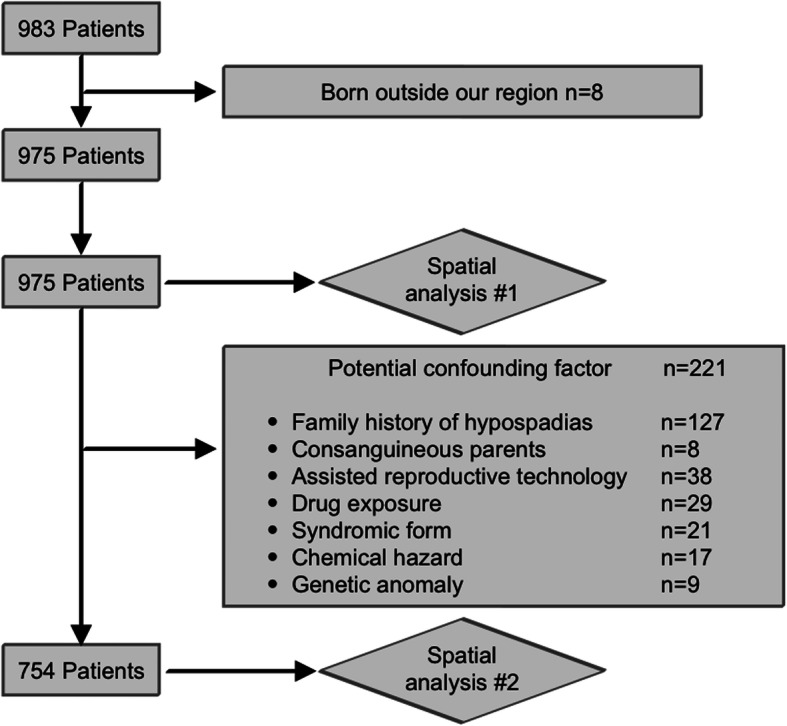
Flow chart for spatial analysis

Of the 983 patients seen in our hospital between January 1999 and December 2012, 8 were excluded for geographical reasons (1 adopted patient, 3 patients born in another region of France, 3 born outside France, and 1 seen at the age of 11 years after referral from another hospital, and for whom zip code at birth was not available) (Fig. 1). The incidence of hypospadias over the 13-year period was 25.4/10,000 male births, and mean annual incidence was 17.67/10,000 male births. Of the 975 included patients, 548 (56.2%) had anterior hypospadias, 319 (32.7%) had middle hypospadias, and 108 (11.1%) had posterior hypospadias. Other data on clinical presentations are summarized in Table 1. We found 221 patients with at least one CF typically associated with a greater risk of hypospadias (as described in section 2.2). These 221 patients were included in layer #1 of our analysis but excluded from layer #2 (Fig. 1).

**Table 1:** Clinical data of all 975 cases included in the spatial analysis. Overall, and stratified on hypospadias form

	Overall	Anterior	Middle	Posterior
n	975	548	319	108
Type (%)				
Anterior	548 (56.2)	–	–	–
Middle	319 (32.7)	–	–	–
Posterior	108 (11.1)	–	–	–
Chordee (%)				
Normal	555 (56.9)	421 (76.8)	127 (39.8)	7 (6.5)
< 45°	202 (20.7)	77 (14.1)	105 (32.9)	20 (18.5)
> 45°	212 (21.7)	49 (8.9)	84 (26.3)	79 (73.1)
NA	6 (0.6)	1 (0.2)	3 (0.9)	2 (1.9)
Hormone treatment (%)				
No treatment	902 (92.5)	537 (98.0)	303 (95.0)	62 (57.4)
Treatment	64 (6.6)	6 (1.1)	13 (4.1)	45 (41.7)
NA	9 (0.9)	5 (0.9)	3 (0.9)	1 (0.9)
Family history (%)				
None	848 (87.0)	488 (89.1)	270 (84.6)	90 (83.3)
1st degree relative	79 (8.1)	39 (7.1)	26 (8.2)	14 (13.0)
2nd degree relative	29 (3.0)	13 (2.4)	13 (4.1)	3 (2.8)
3rd degree relative	19 (1.9)	8 (1.5)	10 (3.1)	1 (0.9)
IUGR (%)	93 (9.5)	33 (6.0)	18 (5.6)	42 (38.9)
Term of delivery (%)				
Term delivery	860 (88.2)	502 (91.6)	295 (92.5)	63 (58.3)
Preterm delivery	113 (11.6)	45 (8.2)	23 (7.2)	45 (41.7)
NA	2 (0.2)	1 (0.2)	1 (0.3)	0 (0.0)
Multiple pregnancy (%)				
Singleton	927 (95.1)	522 (95.3)	308 (96.6)	97 (89.8)
Twins	47 (4.8)	25 (4.6)	11 (3.4)	11 (10.2)
Triplets	1 (0.1)	1 (0.2)	0 (0.0)	0 (0.0)
Age at 1st consultation (months) Median [IQR]	9.48 [4.52;18.69]	11.67 [5.30;23.54]	8.43 [3.98;16.39]	6.64 [3.88;12.47]

*IQR* interquartile range, *IUGR* intrauterine growth retardation*, NA* information not available

**Fig. 2:**
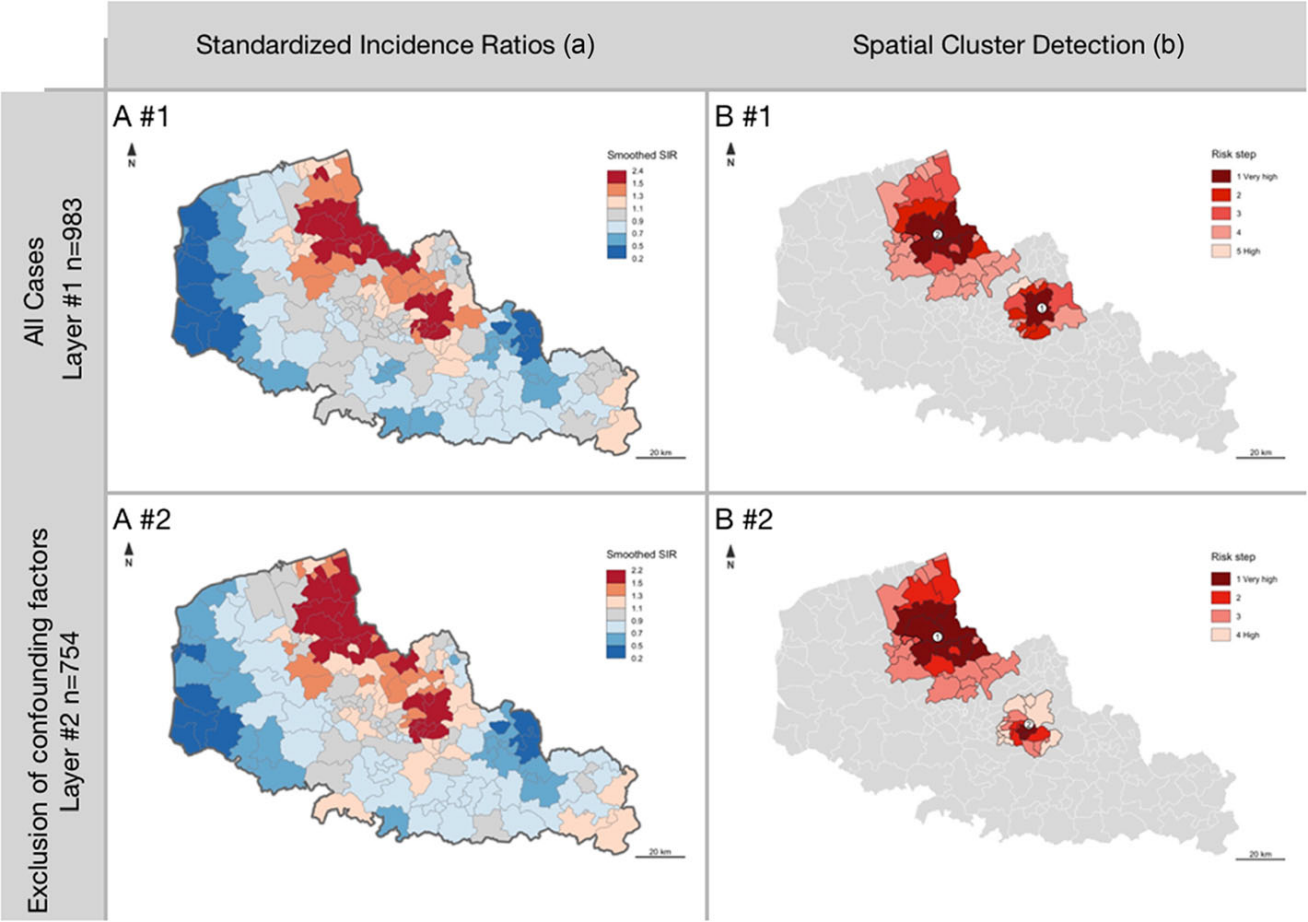
Spatial distribution of age-smoothed Standardized Incidence Ratios (SIR) for hypospadias (1999–2012) in each canton **a** and isotonic spatial cluster detection **b**, for all cases (#1), and after the exclusion of 221 cases with potential confounding factors (#2)

The age-smoothed SIR ranged from 0.2 (95% BCI 0.04, 0.55) to 2.4 (95% BCI 1.29, 3.84) over all cases (layer #1), and from 0.2 (95% BCI 0.04, 0.67) to 2.2 (95% BCI 0.97, 4.15) after exclusion of 221 cases with potential CFs (layer #2) (Fig. 2). In both layer #1 and #2, a Potthoff-Whittinghill test confirmed the spatial variation over the region in the incidence of hypospadias (*p* = 0.005 and 0.008, respectively), and Moran’s test confirmed the spatial correlation (Moran’s index = 0.34, *p* < 0.001 and 0.26, *p* < 0.001, respectively).

The associations between the hypospadias risk and the ecological variables (the French EDI, the proportion of artificialized surface area, the proportion of rural surface area, the proportion of agricultural surface area, and the CWIP) were tested in an ecological regression as shown in the additional figures [see Additional file 1]. None of the associations were statistically significant.

A spatial scan statistic detected two significant isotonic clusters of hypospadias incidence in layer #1 (RR 1.79 and 1.65, *p* < 0.001) and again in layer #2 (RR 1.65 and 1.75, *p* < 0.001) (Table 2, Fig. 2). The clusters characteristics are here described as determined in layer #2 (i.e. after the exclusion of 221 cases with potential CFs). For the cluster characteristics of the first spatial analysis see Additional tables [see Additional file 2].

**Table 2:** Isotonic Cluster characteristics, after exclusion of patients with potential confounding factors

Cluster	*p*	Cluster RR	Population	Isotonic level	Radius (km)	Number of *cantons*	Cases	Expected	RR for each level
1	< 0.0001	1.75	37,368	1	14.8	7	41	15.52	2.83
				2	19.2	3	9	6.3	1.53
				3	28.9	14	67	49.81	1.44
2	< 0.0001	1.65	37,369	1	2.4	2	13	5.39	2.55
				2	6.2	4	37	15.95	2.45
				3	8.3	4	24	19.70	1.29
				4	12.6	9	38	33.99	1.18

*RR* relative risk*, Expected* expected number of cases

The most significant Cluster (#1, “*North-West”*) comprised 24 *cantons* and a total of 37,368 inhabitants, and had an RR of hypospadias of 1.75 (*p* < 0.0001). The second Cluster (#2, “*Center-East”*) comprised 19 *cantons* and a total of 37,369 inhabitants, and had an RR of hypospadias of 1.65 (*p* < 0.0001) (Table 2). It should be kept in mind that the clusters were numbered here in decreasing order of statistical significance; hence, Cluster #1 (*North-West*) determined in layer #2 corresponds to Cluster #2 (*North-West*) determined in layer #1 (Fig. 2).

We also compared clusters with regard to ecological variables and found significant differences for each variable (*p* ≤ 0.001) (Table 3). Cluster #1 (*North-West*) had high proportion of rural surface area (0.90 [0.78; 0.96]), and a high proportion of agricultural surface area (0.85 [0.66, 0.92]), relative to the neutral *cantons* (0.83 [0.52, 0.93] and 0.56 [0.23, 0.70], respectively) and Cluster #2 (*Center-East*) (0.47 [0.36, 0.62] and 0.47 [0.34, 0.54] respectively). In contrast, Cluster #2 (*Center-East*) had a higher proportion of artificialized surface area (0.53 [0.38, 0.64]) and a higher French EDI (i.e. greater deprivation) (0.69 [0.56, 0.73]) than the neutral *cantons* (0.17 [0.07, 0.48] and 0.52 [0.44, 0.65] respectively) and Cluster #1 (*North-West*) (0.10 [0.04, 0.22] and 0.47 [0.42, 0.55] respectively) (*p* < 0.001) did. Lastly, we found a lower distance to the CWIP in Cluster #2 (*Center-East*) (4.92 [2.85, 9.62]).

**Table 3:** Comparison of cantons with regard to ecological data, as a function of presence/absence in each identified high-incidence cluster and after the exclusion of patients with a known potential confounding factor

	Neutral ***cantons***	Cluster #1 (***North-West***)	Cluster #2 (***Center-East***)	***p***
	*N* = 127	*N* = 24	*N* = 19	
French EDI	0.52 [0.44;0.65]	0.47 [0.42;0.55]	0.69 [0.56;0.73]	0.001
Percentage of artificialized area	0.17 [0.07;0.48]	0.10 [0.04;0.22]	0.53 [0.38;0.64]	< 0.001
Percentage of rural area	0.83 [0.52;0.93]	0.90 [0.78;0.96]	0.47 [0.36;0.62]	< 0.001
Percentage of agricultural area	0.56 [0.23;0.70]	0.85 [0.66;0.92]	0.47 [0.34;0.54]	< 0.001
Distance to CWIP (km)	14.4 [7.92;23.6]	12.8 [8.55;16.3]	4.92 [2.85;9.62]	< 0.001

*Note: CWIP* closest waste incineration plant*, EDI* Ecological Deprivation Index*. Statistical comparisons were performed using the Kruskal Wallis test. All results are quoted as the median [IQR]*

With regard to clinical data, we found a significant difference in the preterm rate: the value was 12.2% in neutral *cantons*, 3.42% in Cluster #1 (*North-West*), and 8.93% in Cluster #2 (*Center-East*) (*p* = 0.016) (Table 4).

**Table 4:** Comparison of cantons with regard to clinical data, as a function of presence/absence in each identified high-incidence cluster and after the exclusion of patients with a known potential confounding factor

	Neutral cantons	Cluster #1 North-West	Cluster #2 Center-East	***p***
	*N* = 525	*N* = 117	*N* = 112	
Clinical presentation				
Form, *N* (%):				0.406
Anterior	299 (57.0%)	74 (63.2%)	67 (59.8%)	
Middle	166 (31.6%)	35 (29.9%)	37 (33.0%)	
Posterior	60 (11.4%)	8 (6.84%)	8 (7.14%)	
Chordee, *N* (%):				0.513
None	299 (57.4%)	66 (56.4%)	74 (66.1%)	
< 45°	111 (21.3%)	24 (20.5%)	19 (17.0%)	
> 45°	111 (21.3%)	27 (23.1%)	19 (17.0%)	
Pregnancy				
IUGR, *N* (%):				0.452
No	475 (90.5%)	110 (94.0%)	103 (92.0%)	
Yes	50 (9.52%)	7 (5.98%)	9 (8.04%)	
Preterm delivery, *N* (%):				0.016
No	460 (87.8%)	113 (96.6%)	102 (91.1%)	
Yes	64 (12.2%)	4 (3.42%)	10 (8.93%)	
Multiple pregnancy, *N* (%):				0.892
No	510 (97.1%)	114 (97.4%)	108 (96.4%)	
Yes	15 (2.86%)	3 (2.56%)	4 (3.57%)	
Medical consultation				
Age at 1st medical consultation (months), median [IQR]	10.0 [4.75;20.0]	9.00 [4.00;21.0]	6.00 [3.00;17.0]	0.051
Follow-up (months), median [IQR]	29.0 [20.0;60.5]	29.0 [20.0;63.0]	29.0 [19.0;57.0]	0.75

*Note: IUGR intrauterine growth retardation. Statistical comparisons were performed using the Kruskal Wallis test for quantitative variables and the chi-squared test for qualitative variables*

## Discussion

Our results revealed significant spatial heterogeneity in the incidence of hypospadias and identified two spatial clusters. These results are consistent with previous research performed in northern England, North Carolina, and Nova Scotia [23–25]. When comparing the detected spatial clusters, we found a significant difference in their socio-ecological pattern. The first spatial cluster was characterized by a rural land use pattern, with a higher proportion of rural (and agricultural) land cover, and a lower deprivation index than neutral *cantons* (i.e. less deprived). The second cluster had a more urban and industrial pattern, with higher proportion of artificialized land cover and a higher deprivation index (i.e. more deprived) than neutral *cantons*. It should be noted that the region’s main city (Lille, where our university hospital) fell outside this “urban” cluster. These findings remained significant after exclusion of patients with a known potential CF (i.e. potential bias for spatial analysis). This result reinforces the conclusions of previous spatial analyses of hypospadias - none of which took account of potential CFs [23–25, 28].

Because most of the spatial analysis studies are based on massive administrative datasets, they usually lack clinical information, especially specific information related to the disease of interest such as – in our situation – familial history of hypospadias. In our work, we took into account the clinical information regarding potential CFs associated with a higher risk of hypospadias. Furthermore, the registry included all types of hypospadias - even minor types not requiring surgery (not listed in hospital episodes statistics) and those diagnosed after the child had left the maternity unit (not listed in maternity based birth defect monitoring system).

In the absence of a French national registry of hypospadias cases, we chose to perform a single-center registry study based on the sole referral surgeon for hypospadias in our region’s university hospital (thus in our region). The calculated prevalence was 24/10,000 male births, which was slightly lower than reported data in France with 15.41/10,000 total births (i.e. male and female) [2]. These values suggest that we might have underestimated the regional prevalence of hypospadias; some patients might have been seen by a urologist in a general hospital elsewhere in the region. However, the breakdown in the types of hypospadias was in line with the literature data [44].

We used the zip code at the time of delivery or at the time of the first consultation in our hospital (usually within 12 months of birth for patients with hypospadias) (Table 1). Ideally, we would have analyzed the mother’s zip code before pregnancy and during the first trimester of pregnancy; however, this information was not available retrospectively. Miller et al. showed that 22% of pregnant women moved during pregnancy, and that 51% of these women moved within the same county, [45] and Bell et al. reported that pregnant women typically moved a short distance only (less than 10 km), which would tend not to greatly change their environmental exposure [46].

Some of the environmental factors (or pollutants) reported in the literature are associated to a rural settings (greenhouse workers, pesticides, hazardous waste site proximity etc.), but not all of them are (traffic related pollutants, industry related pollutants, cosmetics etc.) [6, 47]. Spatial analyses can explore the environmental setting as it is. Since collecting high quality ecological data with sufficient granularity is extremely complex (particularly in France), the use of environmental proxys (such as land-use) is an important first step. Abdullah et al. reported on significant spatial clustering among 577 cases of hypospadias in northern England (based on hospital episode statistics), with an association between hypospadias and a lower deprivation index but not with the UK wards’ urban/rural status [25]. Winston et al. showed significant spatial clustering among 995 cases of hypospadias in North Carolina, with a high-risk in areas with > 5% crop cover [24]. And recently, Lane et al. detected significant spatial clustering for hypospadias (and cryptorchidism but none for non-endocrine mediated anomalies such as clubfoot and gastroschisis), and mentioned that their hotspots in Canada were associated with intense agricultural activity but did not underpin this comment with statistical results [23]. In their study, Li et al. found that the prevalence rate was higher in urban areas than in rural areas but that it was increasing more rapidly in rural areas. However, Li et al. did not specify how each area had been classified as either rural or urban [26].

In the present study, there were few differences between the spatial clusters with regard to clinical data. The proportion of preterm births was lower for hypospadias cases from the “rural” cluster (*North-West*) than for cases in the “urban” and most deprived cluster (*Center-East*) and the neutral *cantons*. To put things in perspective, we can observe a higher preterm rate in our hypospadias population compared to the French (general population) data from the Euro-peristat project (11.6% vs 6.6%) [48]. These secondary results should be interpreted with caution, because our hypospadias population is not a random sample of the general population, and our study was built to study the spatial distribution of hypospadias, not preterm birth. Hence environmental factors involved in hypospadias might differ from those involved in preterm birth [6, 49].

## Conclusions

Our results revealed significant spatial clustering in the incidence of hypospadias across our region – even after the exclusion of potential confounding factors – and thus strengthen the findings of previous spatial analyses of this disease. The two identified spatial clusters had significantly different ecological patterns. Our results thus emphasize the complexity of the link between environmental exposure and the incidence of hypospadias; one cannot simply hypothesize that the highest risk occurs in rural areas because of exposure to pesticides.

Our spatial analysis was intended to generate additional medical data on environmental factors and hypospadias. We are of the opinion that spatial analysis and spatial cluster identification could be better used to guide local and regional health policies and to design/guide further observational epidemiological studies on the individual patient level.

## Supplementary information

**Additional file 1 **Ecological regression. This files contains two forest plot representing an ecological regression (see 2.2) comparing the spatial distribution of ecological variables with that of cases of hypospadias: “Additional Figure 1” and “Additional Figure 2”. “Additional Figure 1” represents the ecological regression including all patients (*n* = 975), and “Additional Figure 2” represents the ecological regression after the exclusion of 221 cases with potential CFs (*n* = 754).

**Additional file 2.** Additional tables. This files contains three tables corresponding to the first spatial analysis (i.e. all patients, n = 975) (Additional Table 1, Additional Table 2, Additional Table 3), whereas the main tables displayed in the article corresponds to the second spatial analysis (i.e. after exclusion of 221 cases with potential CFs) (Table 2, Table 3, Table 4). “Additional Table 1” shows the characteristics of the spatial clusters as detected in the first spatial analysis. “Additional Table 2” shows the comparison of cantons with regard to ecological data, as a function of presence/absence in each identified high-incidence cluster, in the first spatial analysis. “Additional Table 3” shows the comparison of cantons with regard to clinical data, as a function of presence/absence in each identified high-incidence cluster, in the first spatial analysis.

## Data Availability

The medical datasets analyzed during the current study are not publicly available due to the sensitiveness of geo-spatial information on each clinical cases, but are available from the corresponding author on reasonable request. National datasets are available on request: CORINE Land Cover: https://www.data.gouv.fr/ Institut National de la Statistique et des Études Économiques: https://www.insee.fr Agence de l’environnement et de la Maîtrise de l’Énergie: https://www.ademe.fr

## Abbreviations

CF: Confounding factor
CWIP: Closest waste incineration plant
EDI: European deprivation index
INSEE: *Institut national de la statistique et des études économiques*
IUGR: Intrauterine growth retardation
SIR: Standardized incidence ratio
