# Supplementary material for: Spatial analysis of hypospadias cases in northern France: taking clinical data into account
Source: BMC Pediatr. 2020 Sep 21;20:442. doi: 10.1186/s12887-020-02332-1 (PMC7504625; doi:10.1186/s12887-020-02332-1)
Supplement: Supplementary file 1 — Additional file 1 Ecological regression. This files contains two forest plot representing an ecological regression (see 2.2) comparing the spatial distribution of ecological variables with that of cases of hypospadias: “Additional Figure 1” and “Additional Figure 2”. “Additional Figure 1” represents the ecological regression including all patients (n = 975), and “Additional Figure 2” represents the ecological regression after the exclusion of 221 cases with potential CFs (n = 754). [file 12887_2020_2332_MOESM1_ESM.docx]

Spatial analysis of hypospadias cases in northern France: Taking clinical data into account.

Arthur Lauriot Dit Prevost^1,2,3 *^, Michael Genin^3^, Florent Occelli^4,5^, René-Hilaire Priso^1,2^, Remi Besson^1,2^, Caroline Lanier^4,5^ and Dyuti Sharma^1,2^.

1 CHU Lille, Clinique de Chirurgie et Orthopédie de l'Enfant, F-59000 Lille, France;  [remi.besson@chru-lille.fr](mailto:remi.besson@chru-lille.fr)

2 CHU Lille, Centre de référence du développement génital DEV-GEN, F-59000 Lille, France; [remi.besson@chru-lille.fr](mailto:remi.besson@chru-lille.fr)

3 Univ. Lille, CHU Lille, ULR 2694 METRICS – Évaluation des technologies de santé et des pratiques médicales, F-59000 Lille, France; [michael.genin@univ-lille.fr](mailto:michael.genin@univ-lille.fr)

4 Univ. Lille, Laboratoire de Génie Civil et géo-Environnement, Lille, F-59000, France : [caroline.lanier@univ-lille.fr](mailto:caroline.lanier@univ-lille.fr)

5 Faculté ILIS/Faculté de pharmacie de Lille – LSVF : [caroline.lanier@univ-lille.fr](mailto:caroline.lanier@univ-lille.fr)

* Correspondence: Clinique de Chirurgie et Orthopédie de l'Enfant, CHU Lille, , F-59000 Lille, France | [arthur.lauriotditprevost@chru-lille.fr](mailto:arthur.lauriotditprevost@chru-lille.fr)

ADDITIONAL FILE 1

Additional Figures


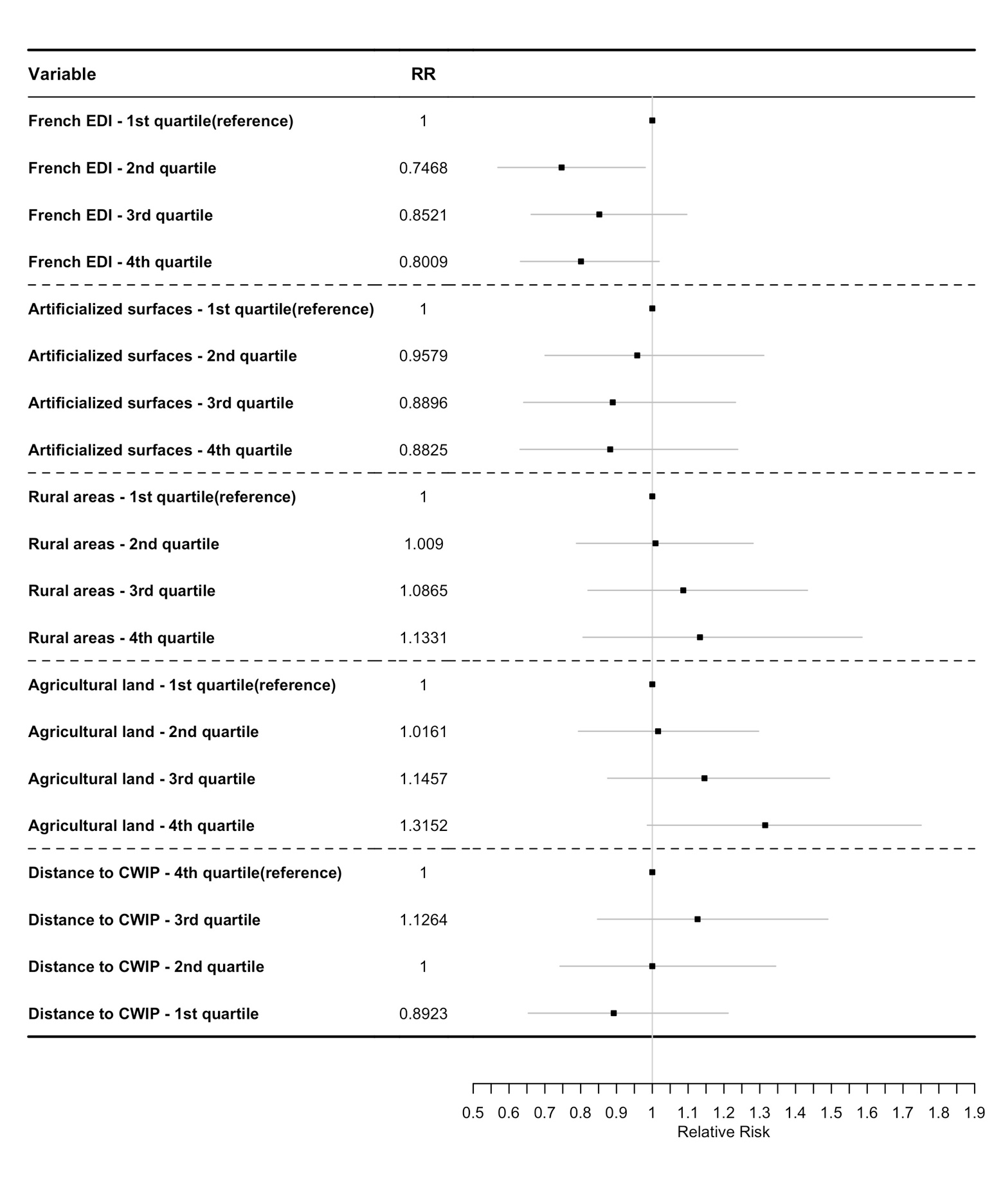


CWIP = closest waste incineration plant, EDI: Ecological Deprivation Index, RR = relative risk.

Additional figure 1. An ecological regression comparing the spatial distribution of ecological variables with that of cases of hypospadias, in the first spatial analysis (All patients, n=975).


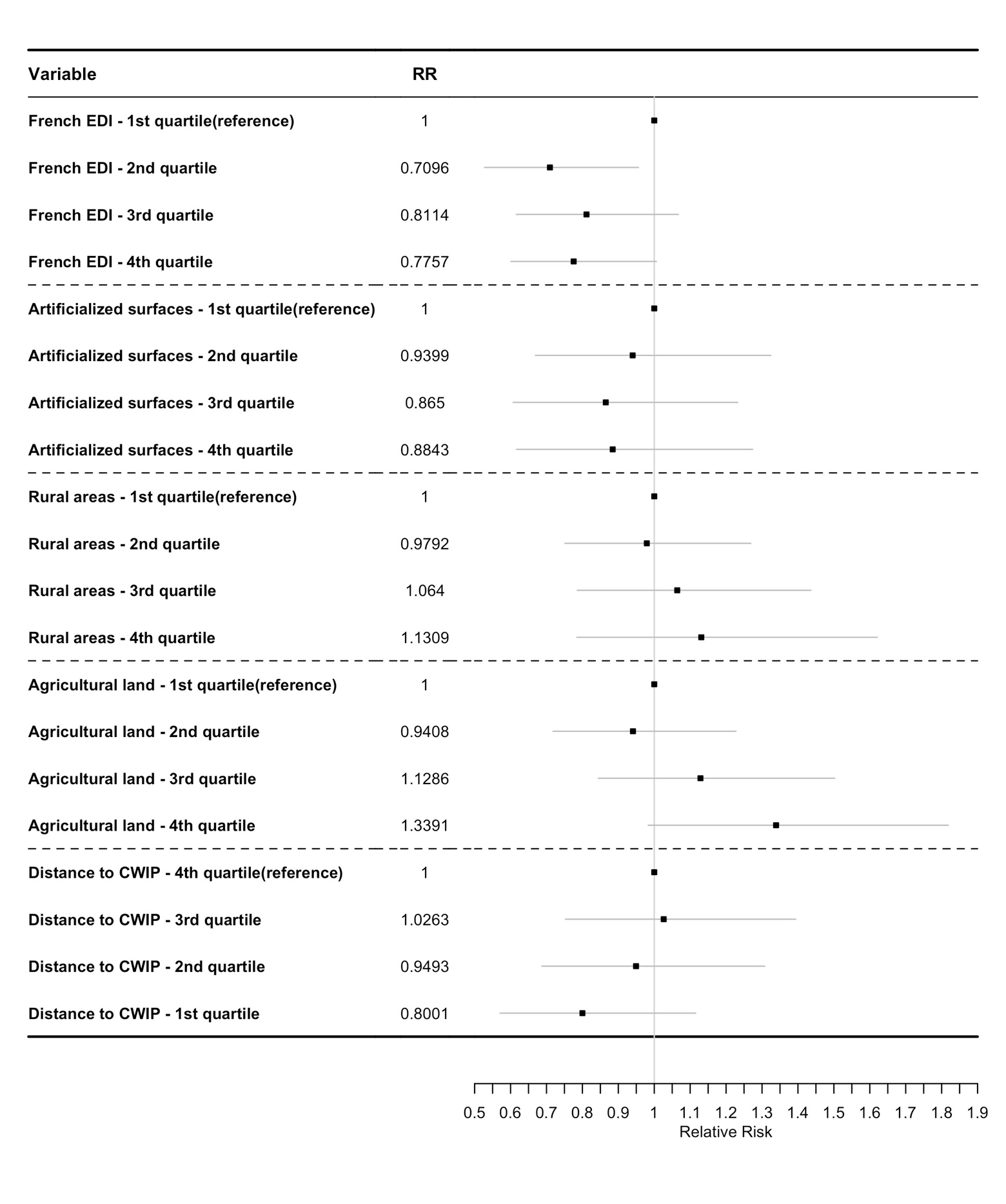


CWIP = closest waste incineration plant, EDI: Ecological Deprivation Index, RR = relative risk.

Additional figure 2. An ecological regression comparing the spatial distribution of ecological variables with that of cases of hypospadias, in the second spatial analysis (after the exclusion of 221 cases with potential CFs, n=754).
