# Supplementary material for: Spatial analysis of hypospadias cases in northern France: taking clinical data into account
Source: BMC Pediatr. 2020 Sep 21;20:442. doi: 10.1186/s12887-020-02332-1 (PMC7504625; doi:10.1186/s12887-020-02332-1)
Supplement: Supplementary file 2 — Additional file 2. Additional tables. This files contains three tables corresponding to the first spatial analysis (i.e. all patients, n = 975) (Additional Table 1, Additional Table 2, Additional Table 3), whereas the main tables displayed in the article corresponds to the second spatial analysis (i.e. after exclusion of 221 cases with potential CFs) (Table 2, Table 3, Table 4). “Additional Table 1” shows the characteristics of the spatial clusters as detected in the first spatial analysis. “Additional Table 2” shows the comparison of cantons with regard to ecological data, as a function of presence/absence in each identified high-incidence cluster, in the first spatial analysis. “Additional Table 3” shows the comparison of cantons with regard to clinical data, as a function of presence/absence in each identified high-incidence cluster, in the first spatial analysis. [file 12887_2020_2332_MOESM2_ESM.docx]

Spatial analysis of hypospadias cases in northern France: Taking clinical data into account.

Arthur Lauriot Dit Prevost^1,2,3 *^, Michael Genin^3^, Florent Occelli^4,5^, René-Hilaire Priso^1,2^, Remi Besson^1,2^, Caroline Lanier^4,5^ and Dyuti Sharma^1,2^.

1 CHU Lille, Clinique de Chirurgie et Orthopédie de l'Enfant, F-59000 Lille, France;  [remi.besson@chru-lille.fr](mailto:remi.besson@chru-lille.fr)

2 CHU Lille, Centre de référence du développement génital DEV-GEN, F-59000 Lille, France; [remi.besson@chru-lille.fr](mailto:remi.besson@chru-lille.fr)

3 Univ. Lille, CHU Lille, ULR 2694 METRICS – Évaluation des technologies de santé et des pratiques médicales, F-59000 Lille, France; [michael.genin@univ-lille.fr](mailto:michael.genin@univ-lille.fr)

4 Univ. Lille, Laboratoire de Génie Civil et géo-Environnement, Lille, F-59000, France : [caroline.lanier@univ-lille.fr](mailto:caroline.lanier@univ-lille.fr)

5 Faculté ILIS/Faculté de pharmacie de Lille – LSVF : [caroline.lanier@univ-lille.fr](mailto:caroline.lanier@univ-lille.fr)

* Correspondence: Clinique de Chirurgie et Orthopédie de l'Enfant, CHU Lille, , F-59000 Lille, France | [arthur.lauriotditprevost@chru-lille.fr](mailto:arthur.lauriotditprevost@chru-lille.fr)

ADDITIONAL FILE 2

Additional Tables

Additional table 1. Characteristics of the spatial clusters as detected in the first spatial analysis (All patients n=975).

| Cluster | p | Cluster RR | Population | Isotonic level | Radius (km) | Number of cantons | Cases | Expected | RR for each level |
| --- | --- | --- | --- | --- | --- | --- | --- | --- | --- |
| 1 | <0.0001 | 1.79 | 33,495 | 1 | 5.9 | 2 | 24 | 11.37 | 2.25 |
|  |  |  |  | 2 | 9.8 | 4 | 45 | 24.94 | 1.93 |
|  |  |  |  | 3 | 10.2 | 2 | 19 | 12.12 | 1.67 |
|  |  |  |  | 4 | 12.1 | 4 | 32 | 21.03 | 1.62 |
|  |  |  |  | 5 | 12.6 | 1 | 19 | 13.57 | 1.49 |
| 2 | <0.0001 | 1.65 | 37,369 | 1 | 14.1 | 5 | 33 | 13.89 | 2.52 |
|  |  |  |  | 2 | 14.8 | 2 | 13 | 6.17 | 2.24 |
|  |  |  |  | 3 | 19.1 | 3 | 12 | 8.14 | 1.56 |
|  |  |  |  | 4 | 28.1 | 14 | 86 | 64.42 | 1.42 |
| *RR: relative risk; Expected: expected number of cases* | | | | | | | | | |

Additional table 2. Comparison of cantons with regard to ecological data, as a function of presence/absence in each identified high-incidence cluster, in the first spatial analysis (All patients n=975).

|  | **Neutral *cantons*** | **Cluster #1 (Center-East)** | **Cluster #2 (North-West)** | **p** |
| --- | --- | --- | --- | --- |
|  | N=133 | N=13 | N=24 |  |
| French EDI | 0.55 [0.44;0.69] | 0.54 [0.40;0.65] | 0.47 [0.42;0.55] | 0.015 |
| Percentage of artificialized area | 0.19 [0.08;0.53] | 0.40 [0.22;0.47] | 0.10 [0.04;0.22] | <0.001 |
| Percentage of rural area | 0.81 [0.47;0.92] | 0.60 [0.53;0.78] | 0.90 [0.78;0.96] | <0.001 |
| Percentage of agricultural area | 0.55 [0.23;0.69] | 0.52 [0.47;0.66] | 0.85 [0.66;0.92] | <0.001 |
| Distance to the CWIP (km) | 12.5 [6.39;22.4] | 14.4 [8.74;19.4] | 12.8 [8.55;16.3] | 0.942 |
| *NB: CWIP = closest waste incineration plant, EDI = Ecological Deprivation Index. Statistical comparisons were performed using the Kruskal Wallis test. All results are quoted as the median [IQR]* | | | | |

Additional table 3. Comparison of cantons with regard to clinical data, as a function of presence/absence in each identified high-incidence cluster, in the first spatial analysis (All patients n=975).

|  | Neutral *cantons* | Cluster #1 (Center-East) | Cluster #2 (North-West) | p |
| --- | --- | --- | --- | --- |
|  | N=692 | N=139 | N=144 |  |
|  | Clinical presentation | | | |
| Type, N (%): |  |  |  | 0.095 |
| Anterior | 376 (54.3%) | 87 (62.6%) | 85 (59.0%) |  |
| Middle | 228 (32.9%) | 44 (31.7%) | 47 (32.6%) |  |
| Posterior | 88 (12.7%) | 8 (5.76%) | 12 (8.33%) |  |
| Chordee, N (%): |  |  |  | 0.04 |
| None | 378 (55.0%) | 95 (68.3%) | 82 (57.3%) |  |
| <45° | 147 (21.4%) | 26 (18.7%) | 29 (20.3%) |  |
| >45° | 162 (23.6%) | 18 (12.9%) | 32 (22.4%) |  |
|  | Pregnancy | | | |
| Multiple pregnancy, N (%): |  |  |  | 0.997 |
| No | 658 (95.1%) | 132 (95.0%) | 137 (95.1%) |  |
| Yes | 34 (4.91%) | 7 (5.04%) | 7 (4.86%) |  |
| IUGR, N (%): |  |  |  | 0.627 |
| No | 622 (89.9%) | 128 (92.1%) | 132 (91.7%) |  |
| Yes | 70 (10.1%) | 11 (7.91%) | 12 (8.33%) |  |
| Preterm, N (%): |  |  |  | 0.028 |
| No | 599 (86.8%) | 125 (89.9%) | 136 (94.4%) |  |
| Yes | 91 (13.2%) | 14 (10.1%) | 8 (5.56%) |  |
|  | Medical consultation | | | |
| Age at 1^st^ medical consultation (months), median [IQR] | 9.00 [4.00;18.0] | 6.00 [3.00;17.0] | 9.00 [4.00;20.0] | 0.12 |
| Follow-up (months), median [IQR] | 29. 0[20.0;59.0] | 28.0 [20.0;54.8] | 29.0 [20.0;64.0] | 0.996 |
|  | Potential confounding factors | | | |
| Family history, N (%): |  |  |  | 0.955 |
| None | 600 (86.7%) | 121 (87.1%) | 127 (88.2%) |  |
| 1^st^ degree relative | 56 (8.09%) | 11 (7.91%) | 12 (8.33%) |  |
| 2^nd^ degree relative | 23 (3.32%) | 3 (2.16%) | 3 (2.08%) |  |
| 3^rd^ degree relative | 13 (1.88%) | 4 (2.88%) | 2 (1.39%) |  |
| Assisted reproductive technology, N (%): |  |  |  | 0.409 |
| No | 664 (96.0%) | 132 (95.0%) | 141 (97.9%) |  |
| Yes | 28 (4.05%) | 7 (5.04%) | 3 (2.08%) |  |
| Drug exposure, N (%): |  |  |  | 0.528 |
| No | 672 (97.1%) | 133 (95.7%) | 141 (97.9%) |  |
| Yes | 20 (2.89%) | 6 (4.32%) | 3 (2.08%) |  |
| Specific chemical exposure, N (%): |  |  |  | 0.793 |
| No | 681 (98.4%) | 136 (97.8%) | 141 (97.9%) |  |
| Yes | 11 (1.59%) | 3 (2.16%) | 3 (2.08%) |  |
| Syndromic presentation, N (%): |  |  |  | 0.884 |
| No | 676 (97.7%) | 136 (97.8%) | 142 (98.6%) |  |
| Yes | 16 (2.31%) | 3 (2.16%) | 2 (1.39%) |  |
| Genetic cause, N (%): |  |  |  | 0.424 |
| No | 685 (99.0%) | 137 (98.6%) | 144 (100%) |  |
| Yes | 7 (1.01%) | 2 (1.44%) | 0 (0.00%) |  |
| *NB: CF = confounding factor. IQR = interquartile range. IUGR = intrauterine growth retardation. Statistical comparisons were performed using the Kruskal Wallis test for quantitative variables and the chi-squared test for qualitative variables.* | | | | |
